# Supplementary material for: Development of a Genome-Informed Protocol for Detection of Pseudomonas amygdali pv. morsprunorum Using LAMP and PCR
Source: Plants (Basel). 2023 Dec 10;12(24):4119. doi: 10.3390/plants12244119 (PMC10747947; doi:10.3390/plants12244119)
Supplement: Supplementary file 1 [file plants-12-04119-s001.zip › Figure S1.pdf]

		1	2	3	4	5	6	7	8	9	10	11	12	13	14	15	16	17	18	19	20	21	22	23	24
CFBP2116	1		88,07	98,73	99,98	88,00	99,99	99,98	99,98	99,96	99,91	99,96	88,01	99,98	99,06	98,73	99,06	88,07	99,98	99,99	99,97	99,94	99,98	99,98	99,98
GCA_000145745.1_ASM14574v1	2	88,07		88,05	88,12	99,97	88,08	88,10	88,13	88,11	88,09	88,12	99,99	88,11	88,13	88,06	88,13	99,99	88,14	88,10	88,14	88,09	88,12	88,12	88,13
GCA_001294215.1_PmpFTRS_U7805	3	98,73	88,05		98,70	88,13	98,70	98,71	98,69	98,68	98,64	98,68	88,08	98,70	98,71	99,98	98,70	88,06	98,73	98,69	98,69	98,68	98,70	98,70	98,69
GCA_001535735.1_ASM153573v1	4	99,98	88,12	98,70		88,15	99,98	99,98	99,98	99,94	99,89	99,93	88,20	99,99	99,03	98,70	99,03	88,13	99,96	99,98	99,96	99,93	99,98	99,97	99,97
GCA_001535755.1_ASM153575v1	5	88,00	99,97	88,13	88,15		88,15	88,12	88,17	88,11	88,10	88,12	99,98	88,14	88,14	88,08	88,13	99,98	88,15	88,12	88,17	88,10	88,10	88,12	88,15
GCA_001535785.1_ASM153578v1	6	99,99	88,08	98,70	99,98	88,15		99,98	99,99	99,94	99,90	99,94	88,19	100,00	99,04	98,70	99,04	88,10	99,96	99,98	99,97	99,94	99,98	99,98	99,98
GCA_002736925.1_ASM273692v1	7	99,98	88,10	98,71	99,98	88,12	99,98		99,98	99,94	99,89	99,93	88,16	99,98	99,03	98,71	99,03	88,11	99,96	99,98	99,96	99,94	99,97	99,97	99,97
GCA_002905685.2_ASM290568v2	8	99,98	88,13	98,69	99,98	88,17	99,99	99,98		99,93	99,88	99,93	88,21	99,99	99,02	98,69	99,01	88,15	99,96	99,97	99,96	99,93	99,98	99,97	99,97
GCA_002905755.1_ASM290575v1	9	99,96	88,11	98,68	99,94	88,11	99,94	99,94	99,93		99,93	100,00	88,16	99,93	99,01	98,68	99,01	88,12	99,95	99,94	99,93	99,92	99,93	99,92	99,94
GCA_002905875.2_ASM290587v2	10	99,91	88,09	98,64	99,89	88,10	99,90	99,89	99,88	99,93		99,93	88,10	99,89	98,97	98,63	98,97	88,11	99,90	99,90	99,88	99,87	99,90	99,88	99,89
GCA_002905895.1_ASM290589v1	11	99,96	88,12	98,68	99,93	88,12	99,94	99,93	99,93	100,00	99,93		88,16	99,93	99,01	98,68	99,01	88,13	99,95	99,94	99,93	99,91	99,93	99,92	99,94
GCA_002916305.1_7968A	12	88,01	99,99	88,08	88,20	99,98	88,19	88,16	88,21	88,16	88,10	88,16		88,18	88,11	88,08	88,11	99,99	88,15	88,20	88,18	88,10	88,11	88,12	88,16
GCA_002916395.1_5270	13	99,98	88,11	98,70	99,99	88,14	100,00	99,98	99,99	99,93	99,89	99,93	88,18		99,04	98,71	99,03	88,12	99,96	99,98	99,97	99,94	99,97	99,97	99,97
GCA_002939205.1_ASM293920v1	14	99,06	88,13	98,71	99,03	88,14	99,04	99,03	99,02	99,01	98,97	99,01	88,11	99,04		98,71	99,98	88,13	99,03	99,04	99,02	99,01	99,03	99,02	99,02
GCA_002939225.1_ASM293922v1	15	98,73	88,06	99,98	98,70	88,08	98,70	98,71	98,69	98,68	98,63	98,68	88,08	98,71	98,71		98,70	88,07	98,73	98,70	98,69	98,69	98,71	98,70	98,69
GCA_002939245.1_ASM293924v1	16	99,06	88,13	98,70	99,03	88,13	99,04	99,03	99,01	99,01	98,97	99,01	88,11	99,03	99,98	98,70		88,13	99,03	99,03	99,02	99,01	99,02	99,01	99,02
GCA_003698945.1_ASM369894v1	17	88,07	99,99	88,06	88,13	99,98	88,10	88,11	88,15	88,12	88,11	88,13	99,99	88,12	88,13	88,07	88,13		88,15	88,12	88,13	88,10	88,12	88,13	88,14
GCA_003700095.1_ASM370009v1	18	99,98	88,14	98,73	99,96	88,15	99,96	99,96	99,96	99,95	99,90	99,95	88,15	99,96	99,03	98,73	99,03	88,15		99,96	99,96	99,91	99,96	99,95	99,96
GCA_003700615.1_ASM370061v1	19	99,99	88,10	98,69	99,98	88,12	99,98	99,98	99,97	99,94	99,90	99,94	88,20	99,98	99,04	98,70	99,03	88,12	99,96		99,95	99,95	99,97	99,96	99,97
GCA_003702205.1_ASM370220v1	20	99,97	88,14	98,69	99,96	88,17	99,97	99,96	99,96	99,93	99,88	99,93	88,18	99,97	99,02	98,69	99,02	88,13	99,96	99,95		99,93	99,96	99,95	99,96
GCA_014653325.1_ASM1465332v1	21	99,94	88,09	98,68	99,93	88,10	99,94	99,94	99,93	99,92	99,87	99,91	88,10	99,94	99,01	98,69	99,01	88,10	99,91	99,95	99,93		99,95	99,93	99,94
S1 Psm	22	99,98	88,12	98,70	99,98	88,10	99,98	99,97	99,98	99,93	99,90	99,93	88,11	99,97	99,03	98,71	99,02	88,12	99,96	99,97	99,96	99,95		100,00	100,00
S2 Psm	23	99,98	88,12	98,70	99,97	88,12	99,98	99,97	99,97	99,92	99,88	99,92	88,12	99,97	99,02	98,70	99,01	88,13	99,95	99,96	99,95	99,93	100,00		99,99
Contigs 11116B2	24	99,98	88,13	98,69	99,97	88,15	99,98	99,97	99,97	99,94	99,89	99,94	88,16	99,97	99,02	98,69	99,02	88,14	99,96	99,97	99,96	99,94	100,00	99,99	

Figure S1. ANIb of the twelve isolates corresponding to *Pseudomonas* species (Table 1) with their respective identity percentage. The strain CFBP2116 (GCA_900289105) was utilized as the reference.
