# Supplementary material for: Development of a Genome-Informed Protocol for Detection of Pseudomonas amygdali pv. morsprunorum Using LAMP and PCR
Source: Plants (Basel). 2023 Dec 10;12(24):4119. doi: 10.3390/plants12244119 (PMC10747947; doi:10.3390/plants12244119)
Supplement: Supplementary file 1 [file plants-12-04119-s001.zip › Figure S2.pdf]

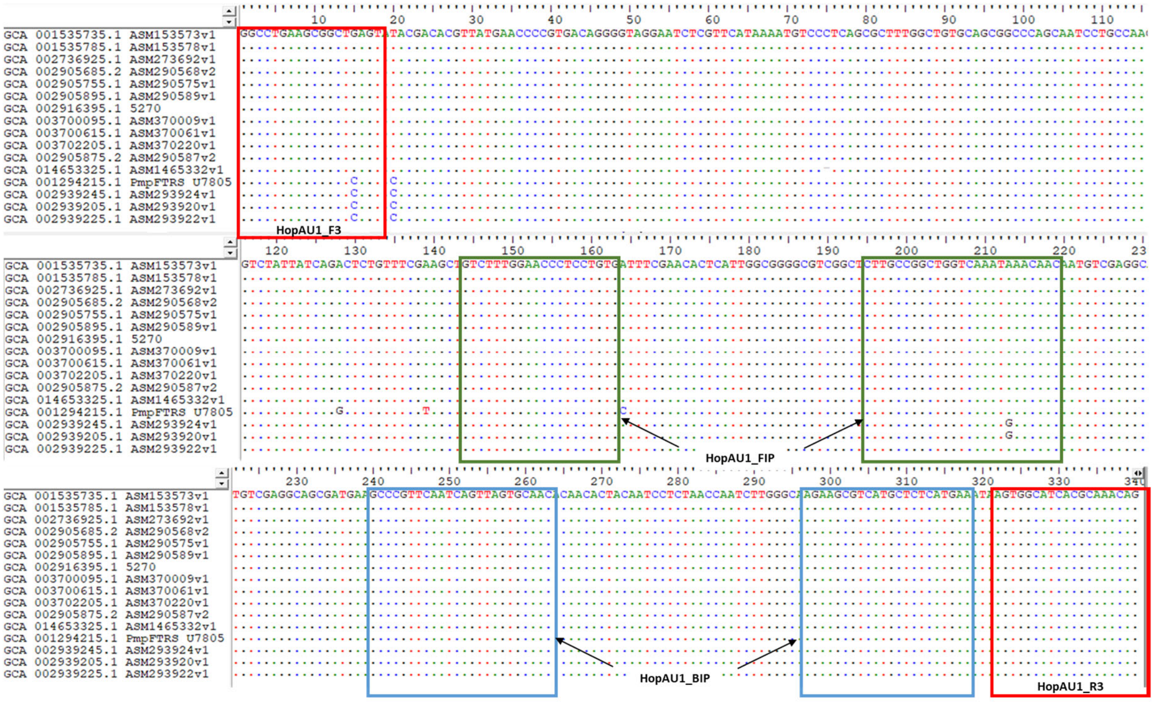

Figure S2. Alignment of the target region used for primers design, extracted from sixteen isolates of *Pseudomonas amygdali* pv. *morsprunorum* available in Genbank. Colored boxes represent the designed primers and are labeled with the corresponding name.
