# Supplementary material for: Development of a Genome-Informed Protocol for Detection of Pseudomonas amygdali pv. morsprunorum Using LAMP and PCR
Source: Plants (Basel). 2023 Dec 10;12(24):4119. doi: 10.3390/plants12244119 (PMC10747947; doi:10.3390/plants12244119)
Supplement: Supplementary file 1 [file plants-12-04119-s001.zip › Figure S3.pdf]

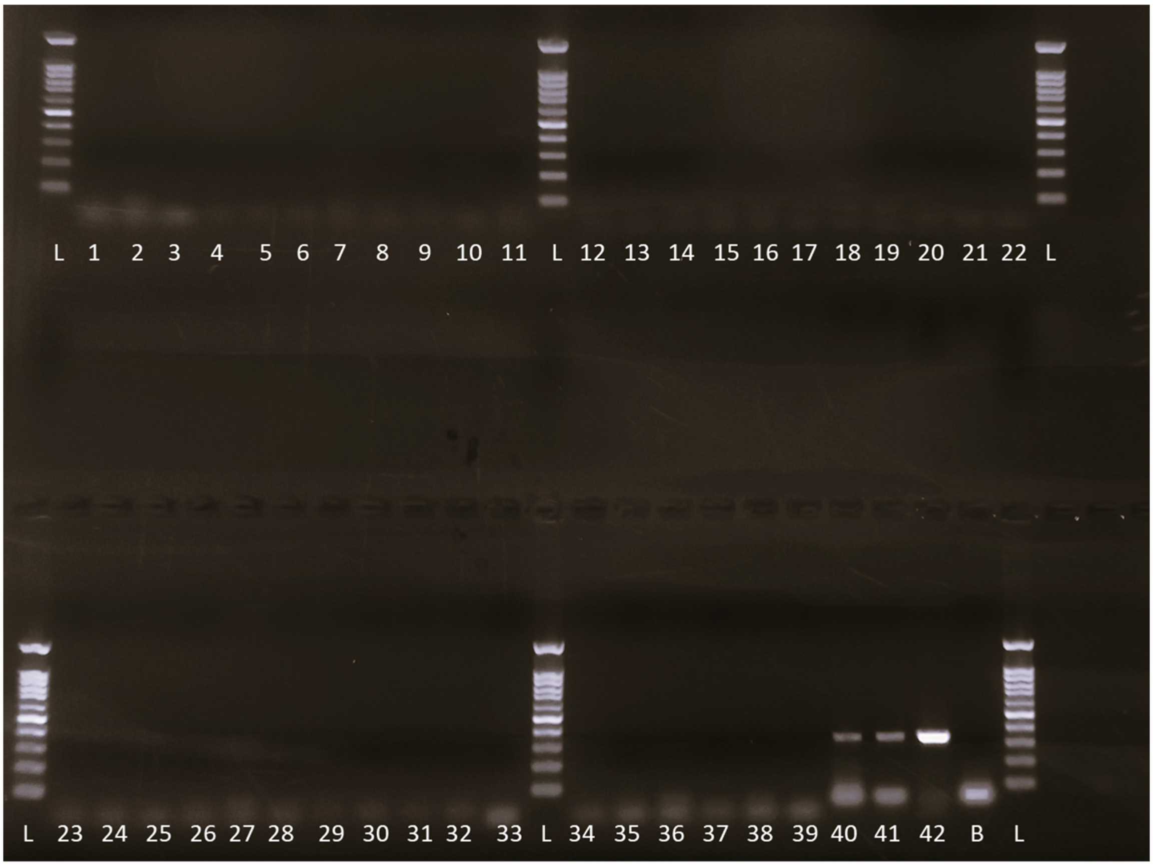

Figure S3. Determination of the specificity of Pam PCR amplification. Samples were loaded as described: (L) 100bp DNA Ladder; Lanes 1-8: *Agrobacterium* sp.; Lane 9: *Xanthomonas* sp.; Lanes 10-39: *Pseudomonas* sp; Lane 40: 11116B2; Lane 41: S1-Pam; Lane 42: S2-Pam; Lane B: Blank control.
