# Supplementary material for: Development of a Genome-Informed Protocol for Detection of Pseudomonas amygdali pv. morsprunorum Using LAMP and PCR
Source: Plants (Basel). 2023 Dec 10;12(24):4119. doi: 10.3390/plants12244119 (PMC10747947; doi:10.3390/plants12244119)
Supplement: Supplementary file 1 [file plants-12-04119-s001.zip › Table S1.pdf]

Table S1. Identification of the seven isolates used for MLSA and ANIb extracted from NCBI.

Isolate Code	Accession Number
PSS B31D	GCA_029763995
P. *savastanoi*	CP076652
P. *amygdali* pv. *tabaci*	NZ_CP042804
CFBP2116	GCA_900289105
P. *avellanae*	NZ_AVEP02000336
P. *viridiflava*	NZ_LT855280
PSS B728	CP000075
