# Supplementary material for: Development of a Genome-Informed Protocol for Detection of Pseudomonas amygdali pv. morsprunorum Using LAMP and PCR
Source: Plants (Basel). 2023 Dec 10;12(24):4119. doi: 10.3390/plants12244119 (PMC10747947; doi:10.3390/plants12244119)
Supplement: Supplementary file 1 [file plants-12-04119-s001.zip › Table S2.pdf]

Table S2. Number of isolates per genera identified in this study.

Genera	Number of Isolates
Pantoea sp.	3
Rosenbergiella sp.	2
Frondihabitans sp	1
Microbacterium sp.	3
Rathayibacter sp.	1
Pseudoxanthomonas sp.	1
Stenotrophomonas sp.	1
Chitinophaga sp.	1
Rahnella sp.	1
Streptomyces sp.	1
Brachybacterium sp.	1
Chryseobacterium sp.	2
Brevundimonas sp-	1
Flavobacterium sp.	1
Staphylococcus sp.	1
Rhizobiaceae sp.	2
Rhizobium sp.	4
Kocuria sp.	2
Bacillus sp.	11
Ensifer sp.	2
Unclassified Rhizobiaceae sp.	1
Xanthomonas sp.	1
Sphingopyxis sp.	3
Pseudomonas sp.	30
Arthrobacter sp.	2
Variovorax sp.	2
Curtobacterium sp.	19
Sphingomonas sp.	21
Terribacillus sp.	2
Agrobacterium sp.	8
